# A Paradigm for Optimal Management: Traumatic Neck Injuries at a Tertiary Care Centre in South India

**DOI:** 10.7759/cureus.81311

**Published:** 2025-03-27

**Authors:** Ravindran Chirukandath, Sumin V Sulaiman, Tinu Sasi, Nanditha Suresh, Agni Vyas R, Gopika Syamsundar, Antony Kiran Mathew

**Affiliations:** 1 General Surgery, Government Medical College, Thrissur, Thrissur, IND

**Keywords:** general trauma surgery, neck vascular injury, penetrating injuries, road traffic injuries, stab injury

## Abstract

Introduction: Traumatic neck injuries represent a critical emergency in surgical practice due to the high concentration of vital structures within the cervical region. These injuries can be classified into blunt and penetrating types, each presenting unique challenges in diagnosis and management. While advances in trauma care have improved patient outcomes, the mortality and morbidity associated with neck injuries remain significant, especially in resource-limited settings.

Materials and methods: A retrospective cohort study was conducted at Government Medical College, Thrissur, analyzing the patterns, management, and outcomes of traumatic neck injuries from January 2022 to December 2024. The study included patients aged 13-79 years, with data collected from medical records, trauma registers, and operative reports. Patient demographics, injury type, anatomical location, management strategies, and clinical outcomes were assessed.

Results: A total of 72 traumatic neck injuries were reviewed, with the majority being young males (n=57, 79%). Blunt trauma (n=45, 62%) was more common than penetrating trauma (n=27, 38%), with road traffic accidents (n=32, 44%) and falls (n=20, 28%) being the leading causes of blunt injuries, while stabbing injuries accounted for most penetrating trauma (n=20, 73%). Emergency neck exploration was required in 17 cases (24%), and the overall mortality rate was seven cases (10%), primarily due to exsanguination (n=5, 67%) and vascular damage (n=2, 33%). The highest mortality was observed in penetrating Zone II injuries (n=5 deaths, 13%), often involving major vascular damage. Complications occurred in 13 survivors (18%), including wound infections (n=6, 8%), pneumonia (n=4, 5%), and neurological deficits (n=4, 5%). Night-time injuries were reported in 43 cases (60%), with a peak incidence during festive seasons and weekends, showing a significant association with alcohol consumption.

Discussion: The findings align with global trends, where penetrating injuries, especially in Zone II, carry a significantly higher risk of mortality. The study underscores the importance of early airway management, rapid surgical intervention, and the role of imaging-based triage for blunt trauma cases. The association of neck trauma with alcohol consumption, especially during nighttime hours, highlights the need for targeted public health measures and enhanced trauma response.

Conclusion: This study provides valuable insights into the epidemiology, management strategies, and outcomes of traumatic neck injuries in a South Indian tertiary care center. The findings emphasize the critical role of early intervention, particularly in penetrating injuries involving vascular structures, and suggest that public health interventions and improved trauma care systems could optimize outcomes in this high-risk population.

## Introduction

Traumatic neck injuries are among the most critical emergencies encountered in surgical practice due to the concentration of vital structures within the confined cervical region. The neck houses major neurovascular bundles, the airway, the esophagus, and the spinal cord, making injuries in this area potentially life-threatening. Even seemingly minor trauma can lead to significant morbidity if underlying damage is not promptly identified and managed [1]. The complexity of these injuries necessitates a meticulous approach to diagnosis, triage, and treatment.

Neck trauma is broadly classified into penetrating and blunt injuries, each with distinct etiological factors and clinical implications [2]. Penetrating injuries, often caused by stab wounds, gunshot wounds, or industrial accidents, carry a higher risk of direct vascular or airway compromise. In contrast, blunt trauma, typically resulting from road traffic accidents (RTAs), falls, or assaults, can lead to airway obstruction, laryngeal fractures, or vertebral injuries with secondary neurological deficits. The decision to intervene surgically depends on multiple factors, including hemodynamic stability, radiological findings, and the presence of hard or soft signs of vascular injury [3].

Despite advancements in trauma care, mortality and morbidity associated with neck injuries remain significant. Penetrating trauma carries a higher immediate mortality risk due to exsanguination or airway obstruction, whereas blunt injuries often lead to delayed complications, including cervical spine instability, airway edema, and neurological impairment. Reported mortality rates vary widely based on injury severity and access to timely medical intervention. In resource-limited settings, delays in diagnosis and surgical intervention further contribute to poor outcomes.

The management of traumatic neck injuries has evolved from a predominantly surgical exploration-based approach to a more selective, evidence-driven strategy [4]. Advances in imaging, particularly CT angiography, have enabled better delineation of vascular and aerodigestive injuries, allowing for nonoperative management in select cases. However, the need for immediate neck exploration remains crucial in patients presenting with active hemorrhage, expanding hematoma, airway distress, or neurological deterioration. Surgical decision-making is often influenced by institutional protocols and available expertise.

In South India, the burden of traumatic neck injuries is rising due to increasing RTAs, interpersonal violence, and workplace accidents. However, regional data on injury patterns, management strategies, and patient outcomes remain limited. Understanding the epidemiology and outcomes of neck trauma in this setting is essential for optimizing treatment protocols and resource allocation.

This study aims to analyze the patterns, mechanisms, and outcomes of neck injuries managed at a tertiary care center in South India over a three-year period. By examining the proportion of penetrating versus blunt injuries, mortality and morbidity rates, and the role of surgical versus conservative management, we hope to provide valuable insights that can guide future trauma care policies and protocols.

## Materials and methods

This retrospective cohort study was conducted in the Department of General Surgery, Government Medical College, Thrissur, a tertiary care center receiving a significant volume of trauma cases. This study was approved by the Institutional Ethics Committee (IEC) of Government Medical College, Thrissur, under IEC number IEC/GMCTSR/2024/235, ensuring adherence to ethical guidelines in the conduct of the research. The objective was to analyze the patterns and outcomes of traumatic neck injuries, differentiating between blunt and penetrating trauma, and to evaluate the anatomical distribution of injuries. The study included all patients aged 13 to 79 years who presented with traumatic neck injuries to the emergency and trauma department between January 2022 and December 2024. Pediatric patients below 13 years and elderly patients above 79 years were excluded.

Patient data were collected retrospectively from operative records, trauma registers, and histopathology reports. Key parameters analyzed included patient demographics (age, sex), injury patterns (blunt vs. penetrating trauma), anatomical location of injuries, management strategies (surgical vs. conservative), and clinical outcomes in terms of morbidity and mortality. The pattern of clinical presentation was also assessed. Data were recorded using a standardized proforma, entered into Microsoft Excel (Redmond, WA, USA), and analyzed using SPSS software (IBM Corp., Armonk, NY, USA). Qualitative variables were expressed as percentages and analyzed using the chi-squared test.

Each case was categorized based on the type of trauma (blunt vs. penetrating), the anatomical zone of injury (Zone I, II, or III), and the management approach (operative vs. non-operative). Imaging modalities, including CT angiography, were used for diagnostic evaluation in stable patients, while hemodynamically unstable patients underwent immediate surgical intervention as per standard trauma protocols. Surgical interventions included neck exploration, vascular repair, tracheostomy, and airway stabilization. Non-operative management involved close monitoring, serial imaging, and conservative measures such as antibiotics and wound care.

Data analysis was conducted using descriptive statistics, with comparisons made between different injury patterns and management outcomes. The study adhered to ethical guidelines, with institutional ethics committee approval obtained prior to data collection. Efforts were made to ensure data accuracy and completeness, though limitations inherent to retrospective studies, such as missing data and selection bias, were acknowledged.

One of the limitations of this study is the potential for incomplete clinical records, which may impact data accuracy. Additionally, as a retrospective analysis, missing data variables could not be controlled.

## Results

A total of 72 cases of traumatic neck injuries were analyzed. The majority of patients were young males (n = 57, 79%), with a mean age of 39.6 years (range: 20-75 years). More than n = 43 (60%) of cases occurred at night, correlating strongly with RTAs and violent assaults. A statistically significant association was observed between nighttime injuries and alcohol consumption (χ² = 12.33, p < 0.001, df = 1, Cramer’s V = 0.414), indicating a strong correlation between alcohol use and nighttime trauma.

**Figure 1:**
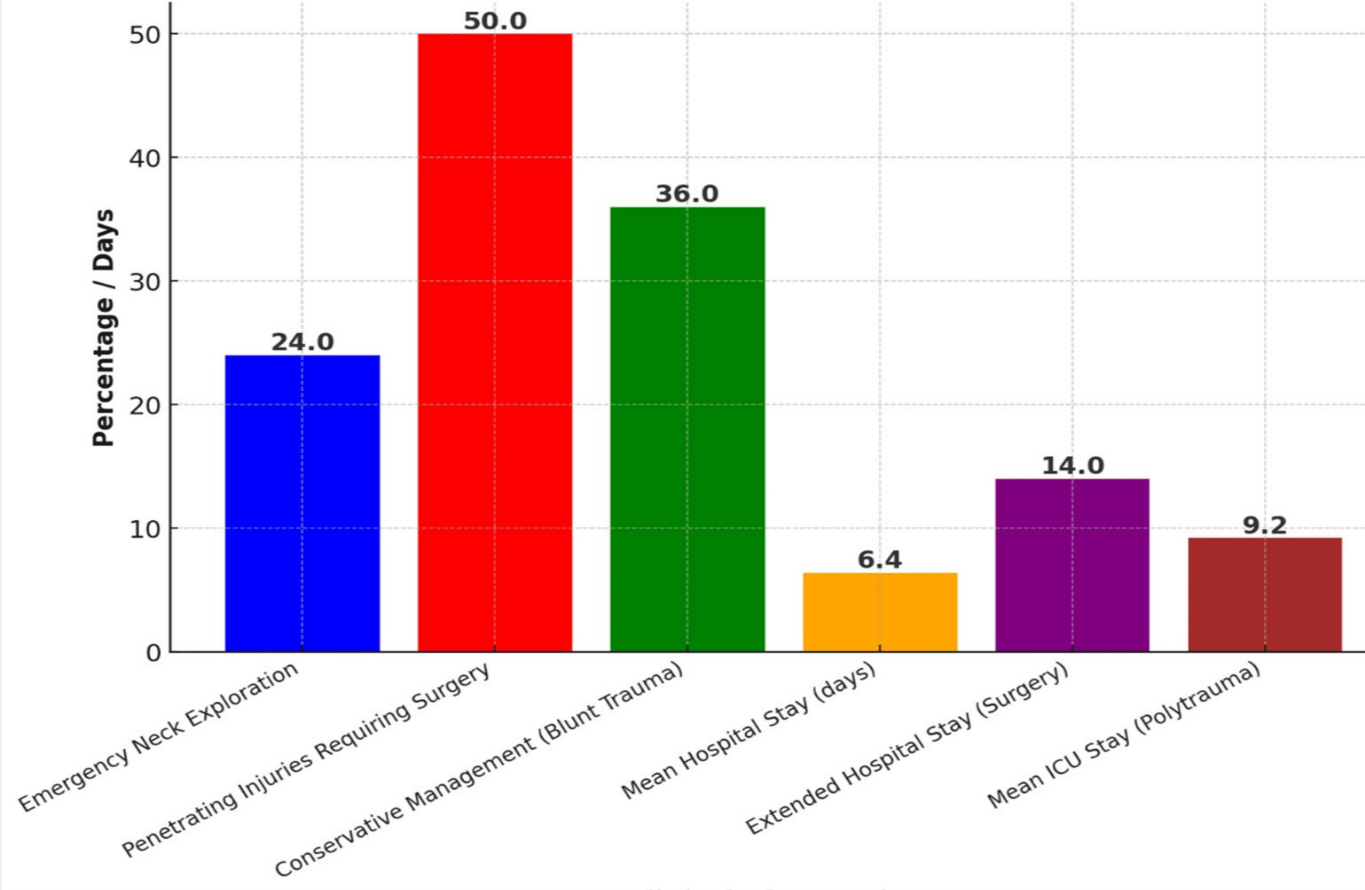
Key Findings in Neck Injury Management

Blunt trauma (n = 45, 62%) was more common than penetrating trauma (n = 27, 38%), but mortality was significantly higher in penetrating injuries. Among blunt trauma cases, RTAs (n = 32, 44%) and falls (n = 20, 28%) were the most frequent causes, whereas stabbing injuries (n = 20, 73%) dominated the penetrating trauma category (Figure 1). One rare case involved a hanging wire wrapping around the neck while riding a bike, resulting in tracheal transection, which required tracheal repair.

Emergency neck exploration was performed in n = 17 (24%) of cases, primarily for expanding hematomas, airway compromise, and suspected major vascular injuries. Among penetrating injuries, n = 13 (50%) required immediate surgical intervention, with vascular repair and tracheostomy being the most common procedures. Conservative management was successful in n = 16 (36%) of blunt trauma cases, particularly those with stable hematomas and minor soft tissue injuries. The mean hospital stay was 6.4 days, but patients requiring surgery had an extended stay of up to 14 days. Prolonged ICU admissions were noted in polytrauma patients and those with associated cervical spine injuries, with an average ICU stay of 5.2 days.

Mortality was observed in n = 7 (10%) of cases, with exsanguination (n = 5, 67%) and vascular damage (n = 2, 33%) being the primary causes. The highest fatality rate was seen in penetrating zone II injuries, where vascular involvement was a major predictor of poor outcomes. A chi-square analysis demonstrated a trend toward higher mortality in penetrating injuries compared to blunt trauma (χ² = 3.56, p = 0.059, df = 1, Cramer’s V = 0.189), suggesting a clinically relevant but not statistically significant difference. Among survivors, n = 13 (18%) developed complications, including wound infections (n = 6, 8%), pneumonia (n = 4, 5%), and neurological deficits (n = 4, 5%). Patients requiring tracheostomy had a higher incidence of pneumonia (p < 0.05).

An interesting seasonal trend was noted, with a peak incidence of neck trauma observed during festive seasons and weekends, likely due to increased travel and alcohol-related incidents. A sub-analysis of patients with alcohol-related injuries revealed that n = 19 (70%) had penetrating trauma, predominantly stab wounds. Comparative analysis with national and international studies suggests that while overall mortality rates align with global data, the proportion of penetrating injuries leading to fatal outcomes was higher than expected, emphasizing the need for rapid intervention in such cases.

A chi-square analysis evaluating the relationship between surgical management and complication rates showed a moderate effect but did not reach statistical significance (χ² = 3.38, p = 0.066, df = 1, Cramer’s V = 0.217), suggesting that complications were not significantly different between surgical and conservative management groups.

In this study of 72 traumatic neck injuries, the distribution of injuries across the different zones of the neck was as follows:

In Zone I (lower neck), n = 15 (20.8%). Blunt trauma was the leading cause, with n = 8 cases due to RTAs, n = 4 to falls, and n = 3 to industrial accidents. Surgical intervention was required in n = 7 (47%), primarily for tracheal or esophageal injuries. The mortality rate in Zone I was 13% (n = 2), with deaths caused by severe respiratory failure and hemorrhage. A chi-square test showed no statistically significant difference in mortality between blunt and penetrating injuries in Zone I (χ² = 2.14, p = 0.143, df = 1, Cramer’s V = 0.229).

In Zone II (mid-neck), n = 45 (62.5%). This zone had the highest proportion of injuries, with n = 25 (56%) from RTAs, n = 12 (27%) from violent assaults, and n = 8 from falls and industrial accidents. Vascular injuries were most common in this zone. Emergency neck exploration was performed in n = 15 (33%), mostly for airway compromise and expanding hematomas. Among penetrating injuries, which dominated in Zone II (n = 20, 73%), all required surgical intervention. The mortality rate in Zone II was 13% (n = 5), primarily due to exsanguination and vascular damage. A chi-square analysis showed a significant association between vascular injury and mortality (χ² = 9.47, p = 0.002, df = 1, Cramer’s V = 0.385).

**Figure 2:**
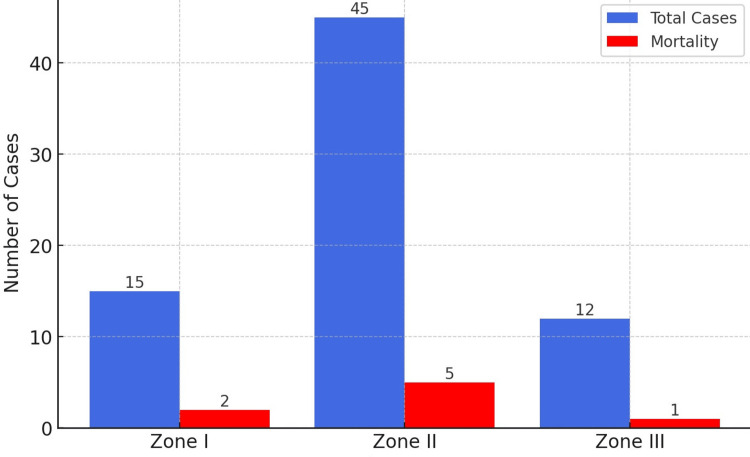
Injury Zones and Mortality Rates

In Zone III (upper neck), n = 12 (16.7%). Violent assaults were the most frequent cause (n = 5, 42%), followed by RTAs (n = 4, 33%). Penetrating trauma dominated (n = 8, 70%), with stab wounds being the predominant injury. Emergency neck exploration was necessary in n = 5 (42%). The mortality rate in Zone III was 8% (n = 1), caused by vascular injury and subsequent hemorrhage. No significant difference was found in mortality rates between surgical and non-surgical cases in Zone III (χ² = 0.84, p = 0.358, df = 1, Cramer’s V = 0.176) (Figure 2).

## Discussion

Neck injuries, whether from blunt or penetrating trauma, present a significant challenge to trauma surgeons due to the complex anatomy and high risk of morbidity and mortality. This study provides a comprehensive analysis of the patterns, management strategies, and outcomes of traumatic neck injuries treated at a tertiary care center in South India over a three-year period.

Demographics and injury patterns

Our findings show that young males (79%) were disproportionately affected, with a mean age of 39.6 years. This aligns with global trauma epidemiology, where young adult males are at higher risk due to greater exposure to RTAs, interpersonal violence, and occupational hazards. The predominance of blunt trauma (62%) over penetrating trauma (38%) mirrors trends seen in other studies from India, Southeast Asia, and Europe, where RTAs remain a leading cause of injury [5].

In contrast to Western data, where firearm-related injuries constitute a significant proportion of penetrating trauma, our study found that stab wounds accounted for 73% of such injuries. This discrepancy reflects differences in social and legal factors governing weapon access. For instance, in the United States, gunshot wounds represent a considerable proportion of penetrating trauma, whereas in European countries, stab wounds are more prevalent [6].

Mortality and morbidity trends

Our study's finding of a higher mortality rate in penetrating neck injuries, particularly those affecting Zone II with vascular involvement, aligns with existing literature [7]. Mortality rates for penetrating neck injuries can reach up to 10%, with vascular injuries being a significant contributor to fatal outcomes. Specifically, arterial injuries occur in approximately 25% of such cases, with carotid artery involvement seen in about 80% of these instances. This underscores the critical importance of early recognition and rapid intervention in managing penetrating neck trauma.

Among survivors, 18% developed complications, with wound infections, pneumonia, and neurological deficits being the most frequent. The presence of neurological deficits in 5% of cases was predominantly observed in patients with cervical spine involvement, underscoring the need for early spinal assessment in neck trauma cases. Prolonged ICU stays were observed in polytrauma patients, further emphasizing the importance of a multidisciplinary approach to trauma management.

Management strategies and surgical outcomes

In our cohort, emergency neck exploration was performed in 24% of cases, mostly for expanding hematomas, airway compromise, and suspected major vascular injuries. This aligns with current trauma guidelines recommending immediate surgical exploration for unstable patients with penetrating injuries, particularly those involving vascular structures. The success of conservative management in 36% of blunt trauma cases, especially those with stable hematomas, reflects a growing trend toward non-operative management in select patients. Recent literature suggests that imaging-based triage, particularly with CT angiography, can help avoid unnecessary surgical interventions while ensuring timely intervention for critical injuries [8].

A key finding in our study was that 80% of penetrating injuries required immediate surgical intervention, with vascular repair and tracheostomy being the most commonly performed procedures. This is in line with data from North American and European trauma centers, where early airway protection and vascular control are paramount in penetrating neck trauma [9]. Our findings further reinforce the role of damage control surgery in cases with severe hemorrhage, emphasizing the need for rapid airway control, hemostasis, and staged surgical interventions in unstable patients [10].

A proportion of patients in our study were managed non-operatively, particularly those with stable hematomas, minor vascular injuries, and soft tissue involvement without airway compromise. These cases were closely monitored with serial clinical assessments and imaging to identify any progression requiring intervention. The success of non-operative management highlights the importance of careful patient selection and the role of imaging in guiding treatment decisions. Our findings align advocate for a more selective approach to surgical intervention, reducing unnecessary procedures while ensuring patient safety and favorable outcomes.

Time trends and seasonal variations

Interestingly, our data revealed a peak in neck injuries during festive seasons and weekends, suggesting a correlation with increased alcohol consumption, travel-related accidents, and interpersonal violence. This pattern has been previously documented in trauma literature, where alcohol-related trauma surges are reported during major holidays [11]. These findings underscore the potential benefits of targeted public health interventions, stricter enforcement of road safety measures, and community awareness programs during high-risk periods.

Another notable trend was the predominance of nighttime injuries (60%), particularly among RTA-related cases. Studies have shown that reduced visibility, fatigue, and alcohol intoxication contribute significantly to nighttime crashes [12]. This highlights the need for improved road lighting, stricter enforcement of driving regulations, and better trauma response preparedness during nighttime hours.

Our study findings are consistent with reports from Indian and global trauma registries, where blunt trauma remains predominant [13], but penetrating injuries carry higher morbidity and mortality. However, unlike Western countries, where firearm-related penetrating injuries are more prevalent, stab wounds and industrial accidents were the leading causes of penetrating trauma in our cohort. This difference highlights the need for region-specific trauma management strategies tailored to local injury patterns.

The success of non-operative management in select blunt trauma cases aligns with a growing body of evidence suggesting that CT angiography-based decision-making can significantly reduce unnecessary surgeries without compromising outcomes [14]. However, our study also reinforces the fact that timely surgical intervention remains the cornerstone of management in unstable penetrating injuries, particularly those with vascular or airway involvement.

The distribution of zones underscores the significant burden of Zone II injuries, which made up the majority of cases, particularly due to the prevalence of penetrating trauma. The association of Zone II injuries with higher mortality due to vascular involvement highlights the critical need for rapid surgical intervention. In contrast, Zone I and Zone III injuries, while less common, still posed significant challenges, particularly in terms of airway management and vascular damage.

Clinical implications and future directions

The findings of this study have several important clinical implications. First, the high mortality in penetrating Zone II injuries suggests a need for more structured trauma protocols, including early vascular imaging and pre-hospital airway management. Second, the high proportion of nighttime and alcohol-related injuries highlights the potential impact of targeted public health interventions, stricter traffic regulations, and enhanced trauma response systems during high-risk periods.

Studies have shown that the use of CT angiography as a first-line imaging modality improves diagnostic accuracy and facilitates timely decision-making, thereby reducing unnecessary surgical interventions [15]. Additionally, advancements in endovascular techniques have provided an alternative to open surgery in select vascular injuries, demonstrating improved outcomes in recent trauma studies [16]. Moreover, the implementation of standardized trauma protocols, including Advanced Trauma Life Support (ATLS) guidelines, has been associated with a reduction in mortality and morbidity in major trauma centers globally [17].

Moving forward, prospective multicenter studies are needed to further evaluate the role of imaging-based triage in blunt trauma cases and refine criteria for non-operative management. Additionally, efforts should focus on improving pre-hospital trauma care, including early airway protection, hemorrhage control, and rapid transport to definitive care centers.

Limitations

Despite its valuable insights, this study has certain limitations. First, as a single-center retrospective study, the findings may not be generalizable to all healthcare settings. Selection bias is a potential concern, as only patients presenting to our tertiary care center were included, potentially underrepresenting cases managed in peripheral hospitals. Additionally, variations in pre-hospital care and transport times may have influenced mortality and morbidity outcomes. The reliance on medical records for data collection introduces a risk of missing or incomplete information. Finally, while statistical analyses were performed to identify associations, causation cannot be definitively established due to the observational nature of the study. Future prospective, multi-center studies with larger sample sizes and standardized data collection protocols are recommended to validate our findings.

## Conclusions

This study provides valuable insights into the patterns and outcomes of traumatic neck injuries in a tertiary care setting in south India. While blunt trauma remains more common, penetrating injuries pose a significantly higher risk of mortality, particularly when involving vascular structures in Zone II of the neck. The findings emphasize the importance of early airway management, rapid surgical intervention, and the role of imaging-based triage in blunt trauma cases. Given the peak incidence of trauma during festive seasons and nighttime hours, targeted public health measures and improved pre-hospital trauma response could further optimize patient outcomes.

The study's limitations include its retrospective design, single-center setting, and potential for selection and reporting bias. Additionally, incomplete follow-up data and variability in trauma management may affect the generalizability of findings.
